# Hepatitis E virus infection among pregnant women in Africa: systematic review and meta-analysis

**DOI:** 10.1186/s12879-019-4125-x

**Published:** 2019-06-13

**Authors:** Mulat Dagnew, Amare Belachew, Moges Tiruneh, Feleke Moges

**Affiliations:** 1 Department of Medical Microbiology, College of Medicine and Health Sciences, School of Biomedical and Laboratory Sciences, University of Gondar, Gondar, Ethiopia; 2 College of Medicine and Health Sciences, Bahir Dar University, Bahir Dar, Ethiopia

**Keywords:** HEV, Infection, Pregnant women, Africa, Systematic review, Meta-analysis

## Abstract

**Background:**

There have been a number of studies about seroprevalence of HEV among pregnant women in Africa. However, the finding of seroprevalence of HEV infection among pregnant women is variable and inconsistent. Therefore; this systematic review intended to provide the pooled seroprevalence of HEV among pregnant women in Africa.

**Methods:**

We searched, Pub Med, Science direct, African online journals and Google scholar electronic data bases and all available references until August 30, 2018. We included cross sectional studies and cohort studies. The search was further limited studies done in African pregnant women. Statistical analysis done by using Stata (version 11) software. The overall pooled prevalence of HEV presented by using the forest plot with 95% CI. The methodological qualities of included studies were assessed using Joanna Briggs Institute Meta-Analysis of Statistics Assessment and Review Instruments.

**Result:**

The pooled seroprevalence of HEV among pregnant women in Africa was 29.13% (95% CI 14.63–43.63). The highest seroprevalence was 84.3% in Egypt and the lowest 6.6% reported in Gabon. There was highest heterogeneity level where I^2^ = 99.7%; *P* < 0.0001.The observed heterogeneity attributed to geographic location/ region, country, assay method used in each study and year of study published. Moreover, HEV seroprevalence varies between countries and within countries. The HEV infection among African pregnant women seems to have a decreasing trend over time.

**Conclusion:**

The seroprevalence of HEV among pregnant women in Africa is high. The seroprevalence of HEV among pregnant women differ with geographic location and assay method. Therefore, it is recommended to conduct further research on commercial ELISA kit sensitivity and specificity, molecular tests, incidence, morbidity and mortality and vertical transmission of HEV from mother to infant in Africa.

**Trial registration:**

CRD42018084963.

**Electronic supplementary material:**

The online version of this article (10.1186/s12879-019-4125-x) contains supplementary material, which is available to authorized users.

## Background

Hepatitis E virus (HEV), first recognized in Asia almost 38 years ago as the main cause of non-A, non-B enterically transmitted hepatitis and the only hepatitis virus that has animal reservoir [1]. HEV is becoming an emerging infectious agent causing mainly acute infection worldwide and a major cause of epidemic water-borne hepatitis in tropical and subtropical countries in areas with poor sanitary conditions. The infection is endemic to southeast and central Asia, the Middle East, and Africa [2].

HEV belongs to a separate family and genus named Hepeviridae and Hepevirus respectively. It is non enveloped, single stranded with positive sense ribonucleic acid (RNA) virus and consists of three open-reading frames (ORFs) [3, 4]. To date, genomic sequence analysis showed that HEV has 7 known mammalian genotypes, of which only four genotypes infect humans, the other genotypes infect animals [5]. Genotype 1 and genotype 2 transmitted by fecal-oral route as water borne disease common in underdeveloped countries and affects humans especially pregnant women [6]. Whereas Genotype 3 and 4 infections associated with sporadic cases and small outbreaks liked to exposure of infected animals and transmitted by consumptions of raw or undercooked meat from infected animals and common in developed countries [7].

According to WHO report, about one third of world population live in areas where HEV is endemic and at risk of infection [8]. HEV infections have occurred in at least 63 countries; about half of these countries have reported large outbreaks [9]. HEV genotypes 1 and 2 account for approximately 20.1 million HEV new infections, 3.4 million cases of symptomatic disease, 70,000 deaths, and 3000 stillbirths [10]. HEV affects primarily young adults and is generally mild; however, the mortality rate is higher among women, especially during the second or third trimesters of pregnancy [11]. Studies from various developing countries have shown that the incidence of HEV infection in pregnancy is high and a significant proportion of pregnant women can progress to fulminant hepatitis, with a mortality rate varying from 30 to 100% [12].

The disease caused by HEV infection is a major public health problem in Africa, especially in resource limited countries. In African countries, a number of HEV outbreaks were reported in Ethiopia, Somalia, Uganda, Democratic republic of Congo, Sudan and South Sudan [13–16]. There was a systematic review of HEV in Africa in all populations [17]. However, there is no systematic review in Africa among pregnant women. There are several studies of HEV among pregnant women available in Africa. The seroprevalence of these studies showed wide variation. Therefore, the objective of such kind of systematic review and meta-analysis is very crucial to scrutinize the burden and pooled seroprevalence of HEV in African pregnant women and inform policy makers and researchers.

## Methods

### Data source and search strategy

We had checked the presence of systematic review and protocol on this topic by searching different data bases. The data bases checked included Cochrane data bases of systematic review, the national health center review and dissemination data base, Joanna Briggs Institute data base a systematic review and implementation of reports (JBI-DSRIR), Health technology assessment (HTA) and the Campbell collaboration library and evidence for policy and practice information (EPPI-center). After checking all the above-mentioned data bases, a comprehensive literature search was conducted starting from September 20–2017-August 30,2018 on PubMed, Science Direct, African Online journal and Google Scholar. The search was carried out by two researchers (MD, FM) independently by using the following key words; name ``Hepatitis E virus seroprevalence``, ``Hepatitis E virus epidemiology`` OR ``Non A non B`` AND ``pregnant women`` AND (Country name ___1 OR Country name_2 OR … ..), where these ellipsis represent names of each African countries. Content experts were consulted for additional materials. The references cited by each eligible study were examined to identify additional articles.

### Eligibility criteria

Cross sectional and cohort studies published in 1993 to August 30, 2018 in Africa were included in the study. There is no language restriction. Articles that assessed the study seroprevalence of HEV infection in pregnant women, studies both HEV and HIV infections on pregnant women, studies on vertical transmission of HEV from mother to infant were included in the study. There was no age restriction. The studies were included only pregnant women population living in Africa.

### Outcome

This review considered studies that include the seroprevalence of HEV infection among pregnant women in Africa as an outcome. The seroprevalence is calculated by dividing the number of HEV infected positive pregnant women over the total number of pregnant women. Seroprevalence is defined as the presence of IgG antibody in serum/plasma of pregnant women by ELISA method.

### Quality assessment

The quality of the studies was assessed using Joanna Briggs Institute (JBI) quality appraisal criteria adapted for studies reporting prevalence data [18]. The following items were used to evaluate prevalence studies: (1) appropriate sample frame; (2) appropriate sampling technique; (3) adequacy of sample size; (4) description of study subjects and setting; (5) sufficient coverage of data analysis; (6) validity of method for identification of condition; (7) standard, reliable measurement for all participants; (8) appropriateness of statistical analysis; and (9) adequacy and management of response rate.

### Data extraction

The relevant data from each selected study have been extracted independently by two authors (MD, FM) and summarized into an excel spread sheet. Discrepancies were resolved through consensus and discussion with a third author (MT). For each selected study, the following parameters were extracted: First author and reference, year of publication, study country/area, year/s/ of study period, study design, IgG prevalence, total number of pregnant women, method employed for HEV detection (ELISA kits).

### Data analysis

Data were analyzed using Stata version 11 software package (Stata Corporation, College Station, TX). A random effects model was used to determine pooled prevalence and the 95% confidence interval (CI), by employing the approach of DerSimonian and Laird [19]. In addition, Freeman Turkey arcsine methodology also used to address stabilizing variances [20]. The heterogeneity of study results was assessed by the use of I^2^ test. Significant heterogeneity was considered for *P* < 0.10 and I^2^ > 50% [21, 22]. Possible source of variation was explored using sensitivity analysis and sub-group analysis by stratifying studies through predetermined variables; study region, type of method used and year of studies published. Publication bias was measured by Begg’s funnel plot and Egger’s regression [23]. A *p*-value < 0.05 on the Egger test was considered indicative of statistically significant publication bias. The forest plot with 95% CI pooled the overall seroprevalence of HEV infection was summarized by using figure. This systematic review and meta-analysis was reported based on PRISMA (Preferred Reporting Items for Systematic Reviews and Meta-Analyses) Statement [24].

## Results

### Study selection

**Fig. 1:**
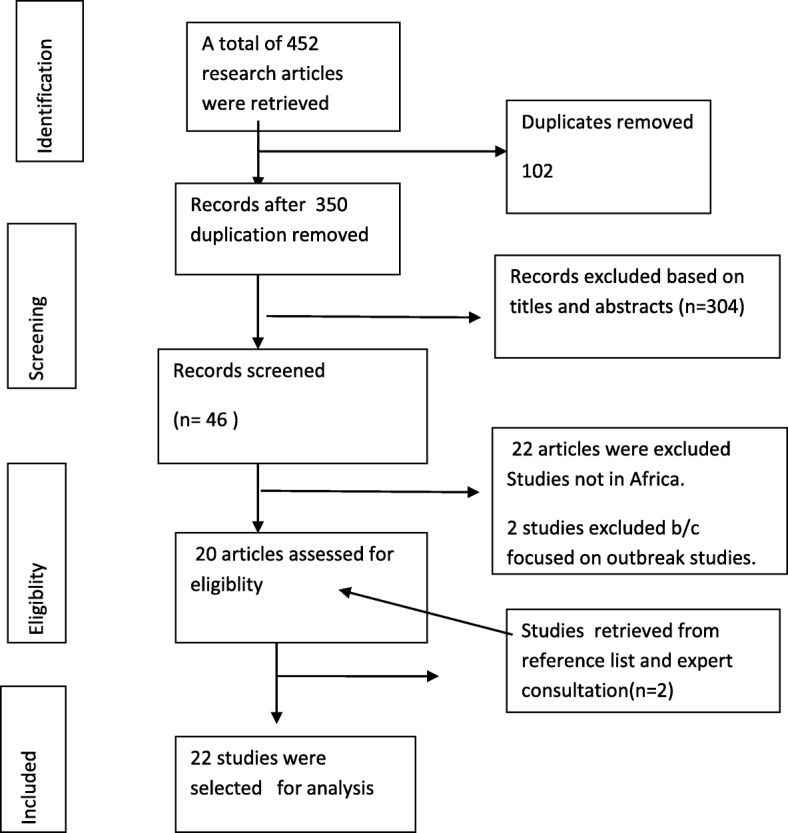
Flow chart shows selection of articles for meta-analysis

A total of 452 research articles were retrieved by electronic search, of these, 350 non-duplicate papers were assessed and 306 records excluded based on titles and study area. The remaining examined by abstract screening of which, 22 articles were excluded because studies had no full text and 2 studies were excluded because they were focused on outbreak studies. After exclusion of duplicates and irrelevant studies based on titles and abstracts, 20 articles were retrieved full text detail analysis. Two additional articles were retrieved from reference list of published articles, finally a total of 22 studies were included in this systematic and meta-analysis (Fig. 1).

### Characteristics of selected studies

The 22 studies included in this systematic review (Table 1) and over all sample size of 8008 pregnant women in Africa [25–46]. The present paper included studies from 12 (21.4%) of the 54 African countries. The regional distribution of countries Eastern Africa included Eritrea, Ethiopia, Tanzania and Sudan. West Africa included Benin, Burkina Faso, Cameroon, Ghana and Nigeria. North Africa included Egypt and Tunisia. Central Africa included only Gabon. The studies were published between 1993 to 2018 and all of the samples collected from 1988 to 2016. The sample size of the selected studies ranged from 90 to 2428. There were large differences in calculated seroprevalence between countries. In the pregnant women, the highest seroprevalence reported from Egypt 84.4% and the lowest seroprevalence from Gabon 6.6%. In addition to the difference in seroprevalences between countries, differences in seroprevalence have also been reported with in countries. Majority of the studies were cross sectional and only two studies were cohort. All studies used ELISA for the diagnosis of HEV. The studies used different types of ELISA assay methods, two studies used Wanti, two studies Dia. Pro, three studies Euroimmun, two studies International immune diagnostics, two studies TMB and other elven studies used different ELISA assay methods (Table 1). Mean age of the studies were specified in 18 studies ranged from 13 years Adje et al. [38] to 50 years Nigussie et al. [34].

**Table 1:** A summary of descriptive characteristics of included studies

Author	Publication year	Study- period	Study design	Country	Age	Sample size (n)	Setting	IgG (%)	Assay method
Depaschal et al. [25]	2016	2014	CS	Benin	15–41	278	Mixed	16.19	Dia. Pro
Traore et al. [26]	2012	2010/2011	CS	Burkina Faso		189	Mixed	11.6	Dia. Pro
Florence et al. [27]	2016		CS	Burkina Faso	18–45	179	Mixed	10.6	Creative diagnostic
Noufensi et al. [28].	2016		CS	Cameroon	16–41	200	Mixed	9	Pristige
Gad et al. [29]	2011		CS	Egypt	15–41	116	Mixed	58.6	Genelabs
Stoszek et al. [30]	2005	1997/2003	CS	Egypt	16–48-	2428	Rural	84.3	In house EIA
El-shety et al. [31]	2014		CS	Egypt	17–40	100	Rural	45	Others
Tekeste et al. [32]	2017	2016	CS	Eritrea	15–49	153	Mixed	26.8	Euroimmun
Abebe et al. [33]	2017	2015	CS	Ethiopia	16–40	386	Mixed	31.6	Wanti
Nigussie et al. [34]	2018	2016	CS	Ethiopia	18–50	846	Mixed	42.4	Wanti
Tsega et al. [35]	1993	1988/91	Cohort	Ethiopia	15–45	32	Mixed	59	Others
Caron et al. [36]	2008	2005	CS	Gabon	14–44	840	Mixed	14.1	TMB
Caron et al. [37]	2012	2008	CS	Gabon	14–43	243		6.6	TMB
Adjei et al. [38]	2016	2008	CS	Ghana	13–42	157	Mixed	28.7	Interna immuno diag.
Obiri-Yeboah et al. [39]	2018	2016	CS	Ghana	28.01	398	Mixed	12.2	Innovita
Junaid et al. [40]	2014	2012	CS	Nigeria	15–40	108	Mixed	25.4	Interna. Immune diag.
Alkali et al [41]	2016	2016	CS	Nigeria	18–45	182	Mixed	9.9	Euroimmun
Lene et al. [42]	2018	2016	CS	Tanzania	27.6	200	Mixed	8	Others
Hannachi et al. [43]	2011	2009	CS	Tunisia	17–52	404	Mixed	12.1	Others
Musa et al	2016			Sudan	15–45	93	Mixed	61.2	Sorono
Al-Tayeb, et al [44]	2014	2013	CS	Sudan	16–42	90	Mixed	41.1	Others
Eltayeb et al. [45]	2015	2013	CS	Sudan	27.5	209	Mixed	12.5	Euroimmun

*CS* cross sectional; Mixed: both rural and urban; Others: Innovita, Pristige,Sorono, Cyproheptadine

### Methodological quality of studies

The JBI criteria’s for assessing the quality of primary studies recommend to include primary studies scored ≥60% of methodological checklists in the meta-analysis (Table 2). We found eight studies scored > 80 (25,27, 28, 34, 35, 42, 44, 46). Six studies scored between 70 and 80% (29,30,31,37,41, 45) and other remaining studies between 60 & 70% (26,32,33,36,38,39,40,43). Almost majority of studies (59%), sample size was adequate, it is greater than 200 (29,31,32,33,39,42,43,45). More importantly, 81% of included studies used ELISA, valid methods for the diagnosis of HEV infection.

**Table 2:** Critical appraisal studies of HEV infection among African pregnant women by using JBI prevalence critical appraisal check list Africa 2018 [18]

Study	Q1	Q2	Q3	Q4	Q5	Q6	Q7	Q8	Q9	Total		
										Y	N	U
Abebe et al. [33]	Y	N	Y	Y	Y	Y	Y	Y	Y	8	1	0
Alkali et al. [41]	Y	N	Y	Y	Y	Y	Y	Y	Y	8	1	0
Adje et al. [38]	Y	N	Y	Y	Y	Y	Y	Y	Y	8	1	0
AlTayeb et al. [44]	Y	N	N	Y	Y	Y	Y	Y	U	6	3	1
Caron et al., 2008 [36]	Y	Y	Y	Y	Y	Y	Y	Y	N	8	1	0
Caron et al., 2012 [37]	Y	Y	Y	N	Y	Y	Y	Y	Y	8	1	0
Depashale et al. [25]	Y	N	Y	Y	Y	Y	Y	Y	Y	7	1	0
El-shety et al. [31]	Y	N	N	N	Y	Y	Y	Y	Y	6	3	0
Eltayeb et al. [45]	Y	N	Y	Y	Y	Y	Y	Y	U	7	1	1
Florence et al. [27]	Y	N	Y	N	Y	Y	Y	Y	Y	7	2	0
Gad et al. [29]	Y	N	N	Y	Y	Y	Y	Y	N	6	3	0
Hannachi et al. [43]												
Junaid et al. [40]	Y	N	N	Y	Y	Y	Y	Y	U	7	2	0
Lene et al. [42]	Y	N	Y	N	Y	Y	Y	Y	U	7	2	0
Musa et al. [39]	Y	N	N	Y	Y	Y	Y	Y	N	6	3	0
Nigussie et al. [34]	Y	N	Y	Y	Y	Y	Y	Y	Y	8	1	0
Noufensi et al. [28]	Y	N	N	Y	Y	Y	Y	Y	U	7	2	0
Obri et al. [39]	Y	Y	Y	Y	Y	Y	Y	Y	U	7	0	1
Stozek et al. [30]	Y	N	Y	Y	N	Y	Y	Y	Y	7	2	0
Tekeste et al. [32]	Y	N	N	Y	Y	Y	Y	Y	U	7	2	0
Traore et al. [26]	Y	N	Y	N	Y	Y	Y	Y	Y	7	2	0
Tsega et al. [35]	Y	N	N	Y	Y	Y	Y	Y	U	7	2	1

*JBI* Joanna briggs institute, *N* no, *U* unclear, *Y* yes

### Result of individual studies

There was an old cohort study conducted from 1988 to 1991 and reported 59% in Ethiopia, Eastern Africa [35]. A study in Egypt, North Africa, reported higher seroprevalence 84.3% among pregnant women conducted on 1997–2003 [30]. In 2005 and 2008 a study in Gabon, central Africa reported 14.1 and 6.6% lowest seroprevalence among African pregnant women respectively [36, 37]. Later on, a study conducted in 2010–2011 in Burkina Faso, Western Africa reported 10.6% [27]. Recently, HEV reported 42.4% in Ethiopia [34] and Ghana [39] 12.2% among HIV positive pregnant women (Table 1).

### Over all pooled seroprevalence of HEV among pregnant women

**Fig. 2:**
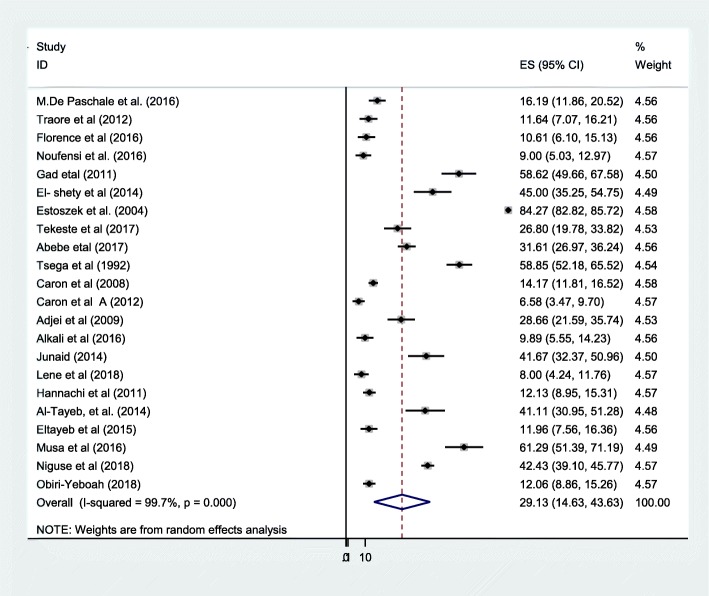
Forest plot of Meta-analysis of HEV in African pregnant women

All 22 included studies were pooled for meta-analysis. As presented on the forest plot (Fig. 2), the seroprevalence of HEV among pregnant women ranged from (6.6–84.27%). The overall pooled seroprevalence of HEV among African pregnant women was 29.13% (95% CI 14.63–43.63). Heterogeneity of studies among reported prevalence using level of heterogeneity was assessed using random effect by I statstic (I^2^ = 99.7%, *P* = 0.001). There was high heterogeneity level. A *p* value of 0.001 indicates the presence of significant heterogeneity and I^2^ = 99.7% indicates the heterogeneity level was high (Fig. 2). To explore the heterogeneity, we have done sensitivity analysis. We further conducted subgroup meta- analysis to identify the source of high heterogeneity by grouping variables: Study country, study region, assay method, year of publication, sample size.

### Sensitivity analysis

**Fig. 3:**
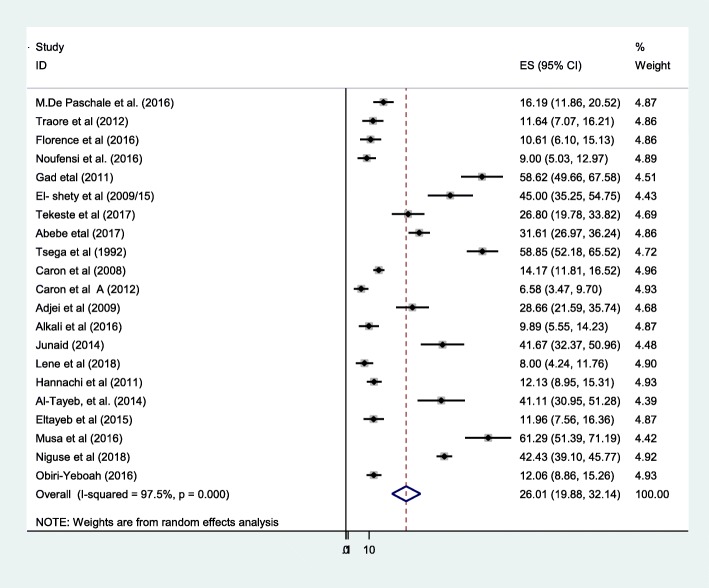
Forest plot of sensitivity analysis of HEV infection among pregnant women

We had done sensitivity analysis by removing one study with large sample size (37). The overall pooled prevalence was 26.01% (95% CI:19.9–32.1) with I^2^ = 97.5%, *P* = 0.0001 (Fig. 3). Additional file 1: Figure S1. However; there was substantial heterogeneity.

### Subgroup analysis

**Fig. 4:**
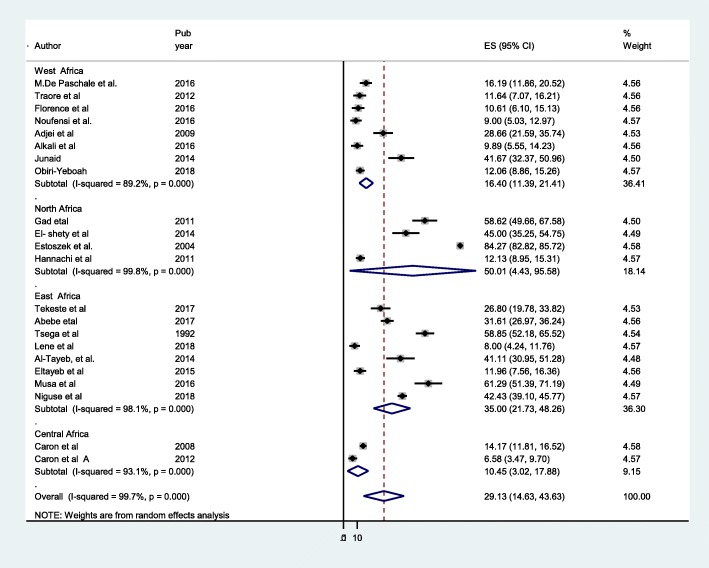
Forest plot of subgroup analysis by subregion of HEV infection among pregnant women

**Fig. 5:**
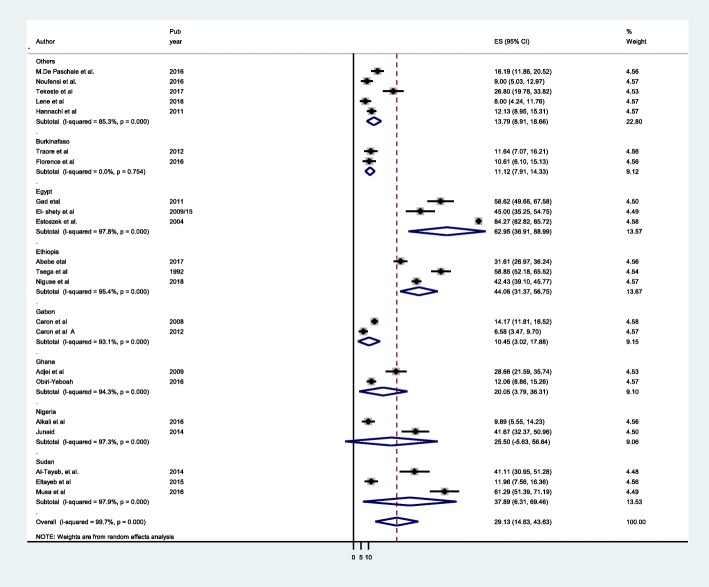
Forest plot of Subgroup analysis by country of HEV infection among pregnant women

Stratified analysis of HEV infection on pregnant women based on different regions of Africa. There was difference of seroprevalence in different regions estimated HEV seroprevalence in pregnant women in West Africa as 16.40 (95% CI 11.39–21.41), North Africa 50.01 (95% (4.43–95.58), East Africa 35.0 (95% CI 21.74–48.26), Central Africa 10.45 (95%, CI 3.02–17.88). There was difference across the region (I^2^ = 99.7, *P* = 0.001) (Fig. 4). In addition to this the North Africa pooled estimate and central Africa are 50.01 and 10.45% respectively, they are out of the overall range (14.63–43.63). Such regional difference has been described between North Africa highest seroprevalence and Central Africa lowest seroprevalence. In addition to differences seroprevalence between countries, there have been also reported differences with in countries. For instance, there have been great differences in seroprevalence in Egypt 45–84.3%, Ethiopia 31.1–58% and Sudan 12.5–61.2% among pregnant women (Fig. 5).

**Fig. 6:**
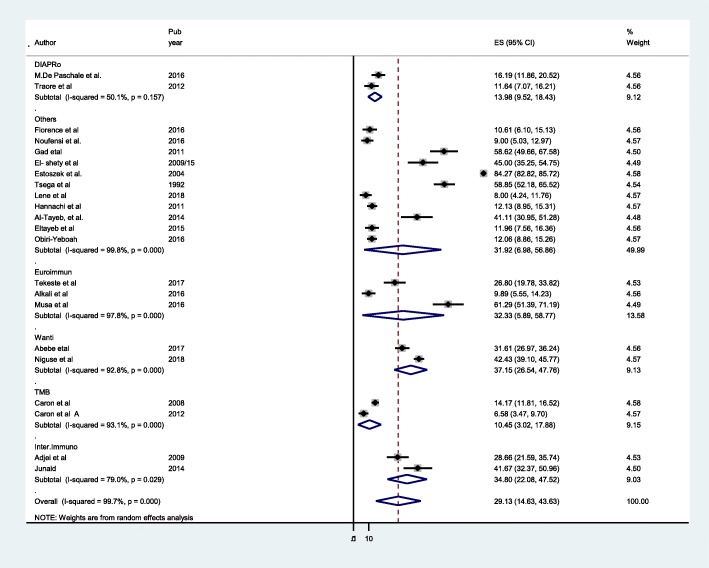
Forest plot of subgroup analysis by assay method of HEV infection among pregnant women

The pooled anti-HEV IgG seroprevalence determined by different commercial ELISA assays showed large variability with reported seroprevalence rates ranging from 10.45 to 32.18 (Fig. 5). The frequently used assays in this systematic review were Euroimmun, Wanti, International immune diagnostics and TMB. For these four assays the pooled seroprevalence rates among pregnant women in Africa were: Wanti 37.15 (26.54–47.76%), International immune diagnostics 34. 80 (22.08–47.52%) Euroimmun 32.33 (5.89–58.77%) and others 31.92(6.98–58.88%) (Fig. 6).

**Fig. 7:**
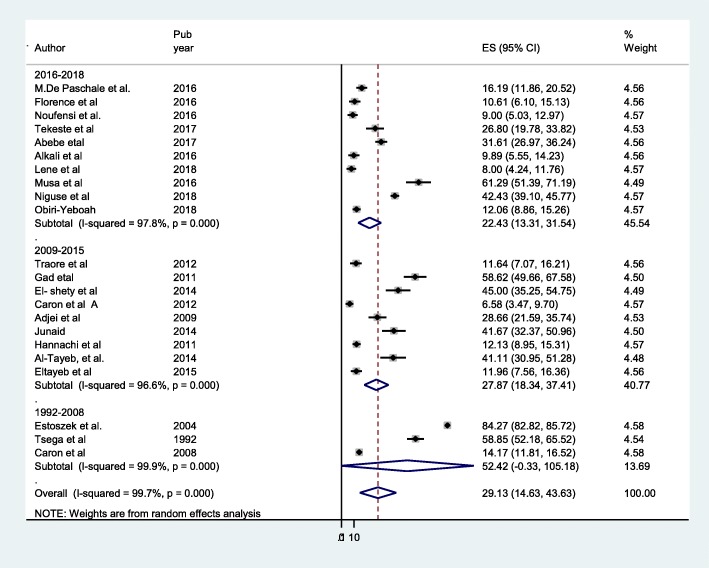
Forest plot of subgroup analysis by year publication of HEV infection among pregnant women

**Fig. 8:**
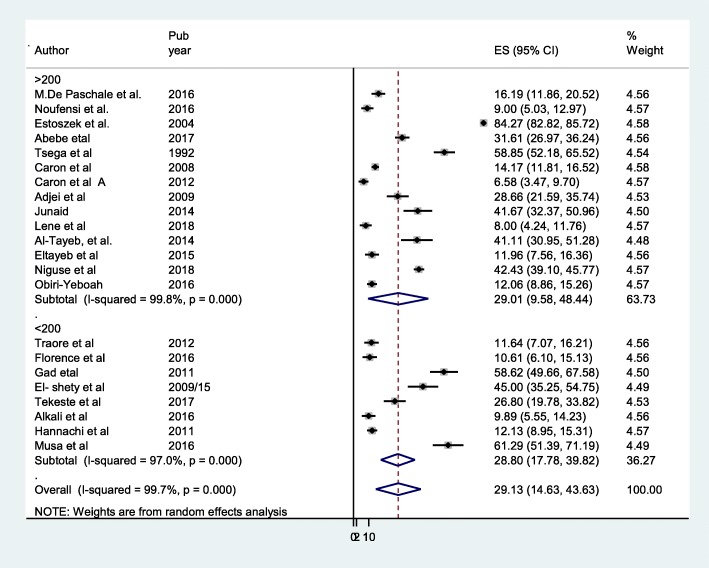
Forest plot of subgroup analysis by sample size of HEV infection among pregnant women

This meta-analysis revealed that seroprevalence of HEV infection among pregnant women differed by publication year (Fig. 7). When we noted the seroprevalence of HEV among pregnant women with time, it decreases from 1992 to 2018. The infection seems to have a decreasing trend over time (Additional file 2: Figure S2). Seroprevalence of HEV infection varies with sample size (Fig. 8).

### Publication bias

**Fig. 9:**
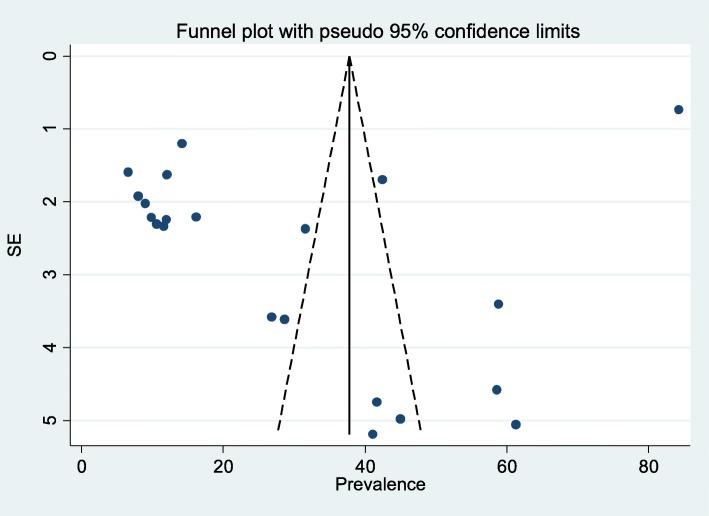
Funnel plot to assess publication bias of studies

We had assessed the publication bias of studies by using funnel plot and Egger’s regression, in this systematic review, there was publication bias, evidence of an Egger’s regression *p*-value< 0.0 was seen when all studies considered (Fig. 9).

Different factors associated with the heterogeneity such as study design, publication date, the sample size of the study, region and assay method were investigated using meta-regression but none of these variables were statistically significant (Table 3).

**Table 3:** Heterogeneity assessment summary by using sensitivity and subgroup analysis

Subgroup analysis	No. of studies	Prevalence of HEV (95%: CI)	Heterogeneity	
			I^2^	*P*
Overall	22	29.1%(14.6–43.6)	99.7%	< 0.00001
Sensitivity analysis by removing one study large sample	21	26%(19.9–32.1)	97.5%	< 0.00001
Sub region of Africa	22			
Central Africa	2	10.5%(3–17.9)	93.1	< 0.00001
East Africa	8	35%(21.7–48.3)	98.1	< 0.00001
North Africa	4	50%(4.4–95.6)	99.8%	< 0.00001
West Africa	8	16.4 (11.4–21.4)	89.2%	< 0.0001
By country having > 2 studies				
Burkina Faso	2	11.1%(7.9–14.3	0	0.754
Egypt	3	63%(37–89)	97.8%	< 0.0001
Ethiopia	3	44 (31.4–57)	95.4%	< 0.0001
Gabon	2	10.5%(3–18)	93.1%	< 0.0001
Ghana	2	20%(4–36)	94.3%	< 0.0001
Nigeria	2	25.5 (5.6–56.6)	97.3%	< 0.0001
Sudan	3	37.9%(6–69.5)	97.9%	< 0.0001
Others	5	13.8%(8.9–18.9)	85.3%	< 0.0001
Publication year				
1993–2008	3	52.4%(.3–105)	99.9%	< 0.0001
2009–2015	9	27.9%(18.3–37.4)	96.6%	< 0.0001
2016–2018	10	22.4%(13.3–31.5)	97.8%	< 0.0001
Sample size				
> 200	14	29 (9.6–48.4)	99.8%	< 0.0001
< 200	8	28.8%(17.8–39.8)	97%	< 0.0001
Assay method				
Dia.Pro	2	14%(9.5–18.4)	50.1%	0.157
Euroimmun	3	32.3 (6–59)	97.8%	< 0.0001
Wanti	2	37.2 (26.5–47.7)	92.8%	< 0.0001
TMB	2	10.5 (3–17.9)	93.1	< 0.0001
Intena.immuno.diag	2	34.8 (22–47.5)	79%	< 0.029
Others	10	31.9%(6.9–56.9)	99.8	< 0.0001

## Discussion

This is the first systematic and meta-analysis review of HEV seroprevalence among pregnant women in Africa. Twenty-two articles comprising a total of 8008 pregnant women were included. Our finding indicates that Hepatitis E virus infection in Africa was high which was ranged from 6.58 Gabon [36] to 84.3% Egypt [30] showing past or current infection of pregnant women in the primary studies included in the review. The discrepancies of HEV seroprevalence among pregnant women in Africa may be, due to geographic location and assay method difference between countries and within countries. Therefore, we have done subgroup analysis by geographic location, assay method and year of publication.

The overall meta-analysis showed that, the pooled seroprevalence of HEV infection among pregnant women in Africa was 29.13% (95% CI, 14.63–43.63; *P* = 0.0001). The findings clearly show that the endemicity of HEV in Africa and burden in particular in pregnant women. A study indicated that HEV infection during pregnancy especially in second and third trimester may lead to hepatic failure and increases risk of mortality 30–100% [12]. Emerging evidences showed that chronic infection, elevated viral load, prolonged viremia have been documented during pregnancy [47, 48]. These contribute to severe liver injury or liver failure in the mother and vertical transmission from mother to infant [49]. Studies indicated that mother to child transmission of HEV infection has been documented and the rate of transmission ranged from 30 to 100% [47, 49].

This finding is in line with systematic review done among blood donors in China 29.2% [50]. However, the finding of this systematic review is higher than a systematic review which was reported 5.4% in Iran among pregnant women [51]. Moreover, our finding is also higher than primary studies conducted among pregnant women in China 11.1% [52], Mexico 5.7% [53], and France 7.7% [54]. However, this finding is lower than a study done in India 60% [55]. The discrepancy may be in Africa and India there is lack of safe drinking water & lack of sanitation. In these areas, 80% of water sewage generated flows in two main water sources: natural rivers, ponds, lakes and underground water. In developing countries, HEV transmitted through fecal-oral route, mainly by consumption of water contaminated by sewerage disposal. In these areas, genotype 1 and 2 are responsible for outbreak in humans [56, 57]. Where as in developed countries, HEV is responsible for sporadic cases due to genotype 3 and 4 that also infect other animals, zoonotic transmission and food- borne transmission [58]. Recent evidences showed that blood transfusion and organ transplant also are the other two transmission pathways in developed countries and these are common in European countries such as Germany and France, Asian region Japan and China and North America countries such as United states [59].

In this systematic review, considerable heterogeneity was found between studies were mainly attributable to sub region/Geographic location, country, HEV anti-IgG ELISA method assay, sample size and the year of study published (Table 3). The subgroup analysis revealed that there was a significant variation among African regions. The highest seroprevalence was seen in North Africa 50.01% (95% CI,4.4–95.6) followed by East Africa 35% (95% CI:21.7–48.3); West Africa 16.4% (95% CI:11.4–21.4) and central Africa 10.5% (95% CI:3.0–17.9). This might be explained that in these two regions there may be high contamination of water by sewerage and lead to high prevalence, epidemic and sporadic of HEV in pregnant women. In line with this, the most common countries frequently hit by HEV epidemic are Algeria, Tunisia, Moroco, Egypt, Somalia,Uganda, Sudan, South Sudan, Kenya and Djibouti are belonged to North and East Africa [14–18].

This review also revealed that, it was not only the difference of HEV seroprevalence in African region but also there was a wide variation of HEV among pregnant women between countries and within countries. The highest seroprevalence reported from Egypt, which was 84.3% [30] and the lowest prevalence reported in Central Africa 6.58% [36]. The difference of HEV seroprevalence among pregnant women between countries and within countries may be difference of sanitary condition, geographical location difference and time difference of study conducted. Surprisingly, HEV infection was the highest in Egypt pregnant women, disease occurs at young age and seroprevalence in this community resembles that of HAV. HEV infection in pregnant women is either asymptomatic or present as mild disease and not fatal or less virulent as compared to other areas because of these reason Egypt classified as distinctive which means from other regions of the world. In relation to this the world HEV epidemiology are four regions: Hyperendemic region, endemic, sporadic and distinctive [60].

When we noted the seroprevalence of HEV among pregnant women with time, it decreases from 1992 to 2018 (Fig. 7). The infection seems to have a decreasing trend over time (Additional file 2: Figure S2), which might be related to improve sanitation and better access of safe potable water in Africa; however, the pattern is not uniform across different countries in Africa. There may be certain confounders such as drought, displacement and war. When we look at some of the countries, HEV seroprevalence decreasing from time to time with in countries in Egypt 84.3% [30] and 58.6 [29], 45% [31] and in Ghana HEV prevalence reported 28.7% [38] and 12. 2% [39], and Nigeria 25.4% [40] and 9.9% [41] with different seroprevalence rates. Further prospective and longitudinal studies on incidence of HEV infection may be required to obtain better knowledge on dynamics of HEV in African continent.

Our subgroup meta-analysis revealed that, the anti-HEV IgG assay used in different study play significant role in the reported seroprevalence rate. In this systematic review, primary studies used more than 12 different commercial assays with different seroprevalence rate (Table 1). The subgroup analysis in (Fig. 5) showed significant heterogeneity among different assay methods employed. This is in line with systematic review and meta-analysis done in Europe countries, seroprevalence rates primarily depend on assays that is employed [61]. Different commercial assays vary significantly in their performance with a large range of specificities and sensitivities [62, 63]. Evidences showed that there were poor concordance b/n assays testing for different antigen epitopes, this indicates, none of the test kits are approved by national health authorities like US Food and drug administration for diagnosis of HEV infection nor validated for estimation of HEV seroprevalence [64, 65]. To this end, studies are lacking in Africa that perform sensitivity and specificity of existing anti- HEV IgG assays. Further studies are recommended in this regard in Africa where HEV is highly prevalent.

Our sensitivity analysis showed that, the overall results had no difference when we excluded one study with large sample size [30]. We have done also sensitivity analysis there is no single study that has beyond the range, the point estimate is 5.65 b/n 4.02 and 7.7. (Additional file 1: Figure S1). The heterogeneity was still substantial (I^2^ = 97. 5%). We also performed subgroup analysis by subregion, assay method employed, publication year and sample size and by country having two or more studies observed reduced and zero heterogeneity (Table 3). However, we have done meta-regression and none of the variables are source of heterogeneity (Table 4). The source of hetrogeniety may be clinical. Therefore, we find out and investigated the source of variation finally performed the pooled estimate. In this systematic review and meta-analysis,there was publication bias, we have detected by using funnel plot and the Egger’s regression *P* < 0.04. The bias may be due to we have used only published studies.

**Table 4:** Meta-regression analysis of variables on HEV infection among pregnant women in Africa

Variables	Coefficient	*P* value
Region	0.601	0.67
Assay method	0.104	0.35
Sample size	0.268	0.15
Country	−0.021	0.51
Year of publication	−0.247	0.13

The implication of this systematic review contributes to understanding of current burden of HEV among pregnant women in Africa, where infectious disease is rampant. To this end, it will significantly contribute the current situation of HEV changing its disease pattern from acute to chronic among pregnant women [47] and vertical transmission of HEV vertically from mother to infant [49]. There is no evidence-based policy in Africa about screening protocol algorithm, treatment guide line and vaccine policies. There is currently a vaccine and highly effective which was approved in China, HEV239 vaccine, the so called Hecolin [66]. Therefore, this systematic review and meta-analysis will provide insights for policy makers, health professionals and researchers to bridge the existing gap.

### Strength and limitation

This study is the first systematic and meta-analysis review among pregnant women in Africa and this meta-analysis has relatively large sample size with good quality studies but also several limitations. The reliability and accuracy of the test depends on the screening method employed. The included studies used different types of ELISA kits for detection of anti- HEV IgG with different specificity and sensitivity. Most studies reported only anti-HEV IgG which shows mostly past infection. In addition, we have included studies more than 24 years of difference. More over almost all studies did not include molecular tests which is important for HEV diagnosis for active infection. Furthermore, data were not available in all 54 African countries, only 12 African countries included in the systematic and meta-analysis.

## Conclusion

Hepatitis E virus infection was high in African pregnant women. Therefore, it is important to screen HEV, conduct further research on commercial ELISA kit sensitivity and specificity, molecular tests, incidence, morbidity and mortality and vertical transmission of HEV from mother to infant in Africa.

## Additional files

Additional file 1: **Figure S1.** Sensitivity analysis of seroprevalence of HEV infection among pregnant women. (DOCX 33 kb)

Additional file 2: **Figure. S2.** Trend of seroprevalence of HEV among African pregnant women. (DOCX 18 kb)

## Data Availability

No additional data required and all information are clearly presented in the main manuscript.

## Abbreviation

ELISA: Enzyme Linked Immuno Sorbent Assay
EPPI: Evidence for policy and practice information
HEV: Hepatitis E virus
JBI: Jonna brings institute
JBI-DSRIR: Joanna briggs institute data base a systematic review and implementation of reports
ORF: Open reading frame
PRISMA: Preferred reporting items of systematic review and meta-analysis
RNA: Ribonucleic acid
