# Supplementary material for: Hepatitis E virus infection among pregnant women in Africa: systematic review and meta-analysis
Source: BMC Infect Dis. 2019 Jun 13;19:519. doi: 10.1186/s12879-019-4125-x (PMC6567642; doi:10.1186/s12879-019-4125-x)
Supplement: Supplementary file 1 — Figure S1. Sensitivity analysis of seroprevalence of HEV infection among pregnant women. (DOCX 33 kb) [file 12879_2019_4125_MOESM1_ESM.docx]

Additional file 1

S1Figure S*ensitivity analysis of seroprevalence of HEV infection among pregnant women*
