# Supplementary material for: Hepatitis E virus infection among pregnant women in Africa: systematic review and meta-analysis
Source: BMC Infect Dis. 2019 Jun 13;19:519. doi: 10.1186/s12879-019-4125-x (PMC6567642; doi:10.1186/s12879-019-4125-x)
Supplement: Supplementary file 2 — Figure. S2. Trend of seroprevalence of HEV among African pregnant women. (DOCX 18 kb) [file 12879_2019_4125_MOESM2_ESM.docx]

Additional file 2

S2 Figure .Trend of seroprevalence of HEV among African pregnant women
